# Feline oral squamous cell carcinoma: recent advances and future perspectives

**DOI:** 10.3389/fvets.2025.1663990

**Published:** 2025-10-13

**Authors:** Paul Tutu, Florentina Daraban Bocaneti, Gennaro Altamura, Mihaela Anca Dascalu, Loredana Horodincu, Octavian Dumitru Soreanu, Oana Irina Tanase, Giuseppe Borzacchiello, Mihai Mares

**Affiliations:** ^1^"Ion Ionescu de la Brad" Iasi University of Life Sciences, Iasi, Romania; ^2^Department of Veterinary Medicine and Animal Productions, University of Naples Federico II, Naples, Italy

**Keywords:** biomarkers, FcaPV, feline, monoclonal antibody, neoplasia, FOSCC

## Abstract

Feline oral squamous cell carcinoma (FOSCC) is the most common oral malignancy in cats, characterized by aggressive local invasion, high metastatic potential, and poor clinical outcomes. Its etiology is multifactorial, involving genetic mutations (notably TP53), viral infections (such as papillomavirus), environmental exposures to xenobiotics and chronic oral inflammation, though definitive causal relationships remain unclear due to limited studies. FOSCC primarily affects older, non-pedigree cats, with no clear sex or breed predisposition, and most frequently arises in the gingiva, sublingual region, and tongue. FOSCC presents with non-specific signs like weight loss, oral ulceration, and difficult eating, often leading to late diagnosis. FOSCC displays highly infiltrative growth with marked cellular pleomorphism and frequent bone invasion. Recent advances have identified various biomarkers, such as Ki-67, Cyclin D1, Bmi-1, and EMT-related proteins, that enhance diagnostic accuracy and prognostic assessment, while emerging research into tumor mutational burden and metabolic pathways offers promising therapeutic targets. Prognosis remains poor, with median survival times typically under 2 months and limited response to conventional treatments; however, surgical intervention and novel targeted therapies show potential for improved outcomes. This review synthesizes recent progress in understanding FOSCC etiology, pathology, and therapeutic strategies, and highlights ongoing challenges and future directions in the management of this devastating feline cancer.

## 1 Introduction

Cancer poses serious health challenges in domestic animals, with feline oral squamous cell carcinoma (FOSCC) being the most common and aggressive oral cancer in cats. FOSCC exhibits rapid local invasion and metastasis, driven by complex genetic, structural, environmental, and infectious factors. Advances in veterinary oncology have revealed key molecular mechanisms and biomarkers, enabling new targeted and immunotherapeutic strategies (1–8). Despite progress, gaps remain in understanding specific risk factors and treatment resistance (9–11). This review emphasizes recent advances in FOSCC research in terms of etiology, epidemiology, prognosis, pathology and explores future perspectives, including new therapeutic approaches and molecular diagnosis, that could further enhance understanding and treatment of this challenging feline cancer.

## 2 Etiology

### 2.1 Viral infection

Several viruses, including FcaPV (*Felis catus* papillomavirus), FIV (Feline immunodeficiency virus), FeLV (Feline leukemia virus), and EBV (Epstein–Barr virus) have been investigated for their role in FOSCC (10–17).

Feline papillomaviruses have been detected in tumor samples, particularly *Felis catus* papillomavirus type 2 (FcaPV-2) (9, 13–15, 17–25). The viral oncoproteins E6 and E7 contribute to oncogenesis by disrupting key tumor suppressor pathways, specifically p53 and pRb, thereby promoting cancer development (22, 26, 27). High viral DNA loads correlate with elevated E6 and E7 expression, indicating that FcaPV-2 can actively drive tumor growth in affected cases (28–31). The overexpression of p16, a surrogate biomarker linked to E7-mediated pRb disruption, may be involved in FcaPV-related FOSCC, although its exact role requires more investigation (13, 17, 25). Disruption of the viral E2 gene, which normally regulates viral transcription, leads to unchecked expression of these oncogenes (22). Notably, the occasional co-expression of L1 capsid protein alongside E6/E7 in tumors suggests ongoing viral replication, maintaining a persistent immune response that fosters a pro-tumorigenic inflammatory microenvironment through tissue damage and cytokine release (25, 28, 32). Interestingly, recent studies indicate that different FcaPV types are detectable in *in situ* carcinoma of the oral cavity, suggesting a viral-driven multi-step carcinogenesis and providing additional evidence of their role in FOSCC development (33). Due to pathological similarities with human head and neck squamous cell carcinoma (HNSCC) associated with high-risk human papillomavirus infection, FcaPV-positive FOSCC is proposed as a relevant animal model for HPV-driven HNSCC (34–37).

FIV, a lentivirus causing immunosuppression similarly to human HIV, infects the oral cavity, creating a viral reservoir that promotes chronic inflammation and immune dysfunction. This environment facilitates cellular dysregulation, increasing neoplastic risk and contributing to FOSCC development (16). The immunosuppressive effects of FIV promote repeated cellular turnover and damage, critical steps in carcinogenesis (38).

FeLV, a retrovirus known for causing lymphomas and sarcomas, may also contribute to FOSCC through insertional mutagenesis, impairing oncogenes and tumor suppressor genes and triggering malignant transformation (39, 40). Therefore, FeLV-induced immune dysregulation and chronic inflammation may further increase susceptibility to FOSCC.

Additionally, EBV has been detected in one FOSCC case, but further research is needed to understand better the possible role of this virus in the etiopathogenesis (12, 41).

Dated studies presented conflicting results regarding detection of viral infection in FOSCC. Variability in sample size and viral detection methods may justify this apparent inconsistency with most recent research. Techniques differ widely, from PCR-based viral DNA detection and immunohistochemistry to *in situ* hybridization, each with varying sensitivity and specificity. Standardization of methodological approaches represents a significant challenge to be addressed in future research in order to definitely clarify the role of viral infections in FOSCC etiology.

### 2.2 Environmental and lifestyle factors

Exposure to environmental tobacco smoke (ETS) has been investigated as a risk factor for FOSCC. However, studies have not found a statistically significant correlation, despite some suggesting a twofold increased risk in exposed cats (1–3, 42). Unlike in humans, no dose–response relationship was observed between exposure to cigarette smoke and cancer development (1–3, 42). Dietary factors have also been investigated, with wet food consumption, especially canned tuna, being associated with a 3.6-fold increased risk. This association may be due to nutrient differences in these foods or because high canned food intake leads to poor dental hygiene, promoting tartar buildup, bacterial toxins, oral inflammation, and potentially neoplastic transformation, though statistical significance was not established (1, 2).

The use of ectoparasite control methods, such as flea collars, has been linked to an increased risk (5.3-fold), where chronic exposure to chemical compounds in these products may induce cellular damage, oxidative stress, or immune disruption, potentially contributing to carcinogenesis, but evidence remains inconclusive (1, 2). Clumping clay cat litter and flea collar use were reported as significant risk factors (ORs 1.66 and 4.48, respectively), possibly related to carcinogenic substances such as crystalline silica in clay litters and tetrachlorvinphos in flea collars (43). These findings suggest environmental chemical exposures may play a role in FOSCC development and warrant further research.

### 2.3 Chronic inflammation and comorbidities

Chronic oral conditions such as periodontal disease (PD), feline chronic gingivostomatitis (FCGS), and other oral inflammatory conditions may contribute to carcinogenesis, though studies directly linking them to FOSCC are lacking (9, 44–47). In humans, chronic inflammation is known to induce genetic mutations and epigenetic alterations, leading to cancer (48). The involvement of inflammatory mediators like cytokines, prostaglandins, and metalloproteinases, which promote tumor progression, has been demonstrated in both human and feline SCC (5, 7, 48–51). Additionally, a case of *Trichinella* spp. infection was reported in an FOSCC sample, but its carcinogenic role remains unclear, further research is required (44, 52).

### 2.4 Genetic and molecular events

Genetic mutations, particularly in the TP53 gene, are commonly found in FOSCC and are thought to play a critical role in tumor development (2, 17, 25, 53, 54). Increased expression of the tumor suppressor protein p53 has been observed in some ETS-exposed cats, no direct link to tobacco exposure was confirmed (2, 3). The overexpression of p16, a biomarker for cell senescence, has been assessed in several studies, but no statistically significant correlation with papillomavirus infection was found, more studies are needed in this area (13, 17, 20, 25, 31, 55, 56). Other molecular pathways, such as cyclooxygenase (COX), signal transducer and activator of transcription 3 (STAT3), epidermal growth factor receptor (EGFR), and vascular endothelial growth factor (VEGF), have been implicated in tumor progression, suggesting a complex interplay of molecular alterations in FOSCC development (5, 7, 49, 51, 57).

### 2.5 Development and structural factors

It has been proposed that FOSCC may originate from dental structures, such as the dental lamina and enamel organ epithelium, similar to dentigerous cyst-associated SCC in humans (58, 59). While this hypothesis remains speculative, it aligns with observations in other species, suggesting a possible developmental contribution to the disease.

### 2.6 Microbial influences and oral flora

Human studies show that imbalances in oral bacteria can promote oral cancer by increasing inflammation (60). In cats, infection with FIV is associated with harmful shifts in oral bacteria, increasing the risk of oral squamous cell carcinoma (61). Differences in oral microbiota between healthy cats and those with periodontitis indicate that bacterial imbalance may also raise cancer risk in felines, paralleling findings in humans (62).

The influence of oral microbiota on the development of FOSCC is an area of ongoing research. Exploring the role of microbial profiles in oral health and disease could identify specific pathogens as potential risk factors for feline oral cancer.

**Figure 1:**
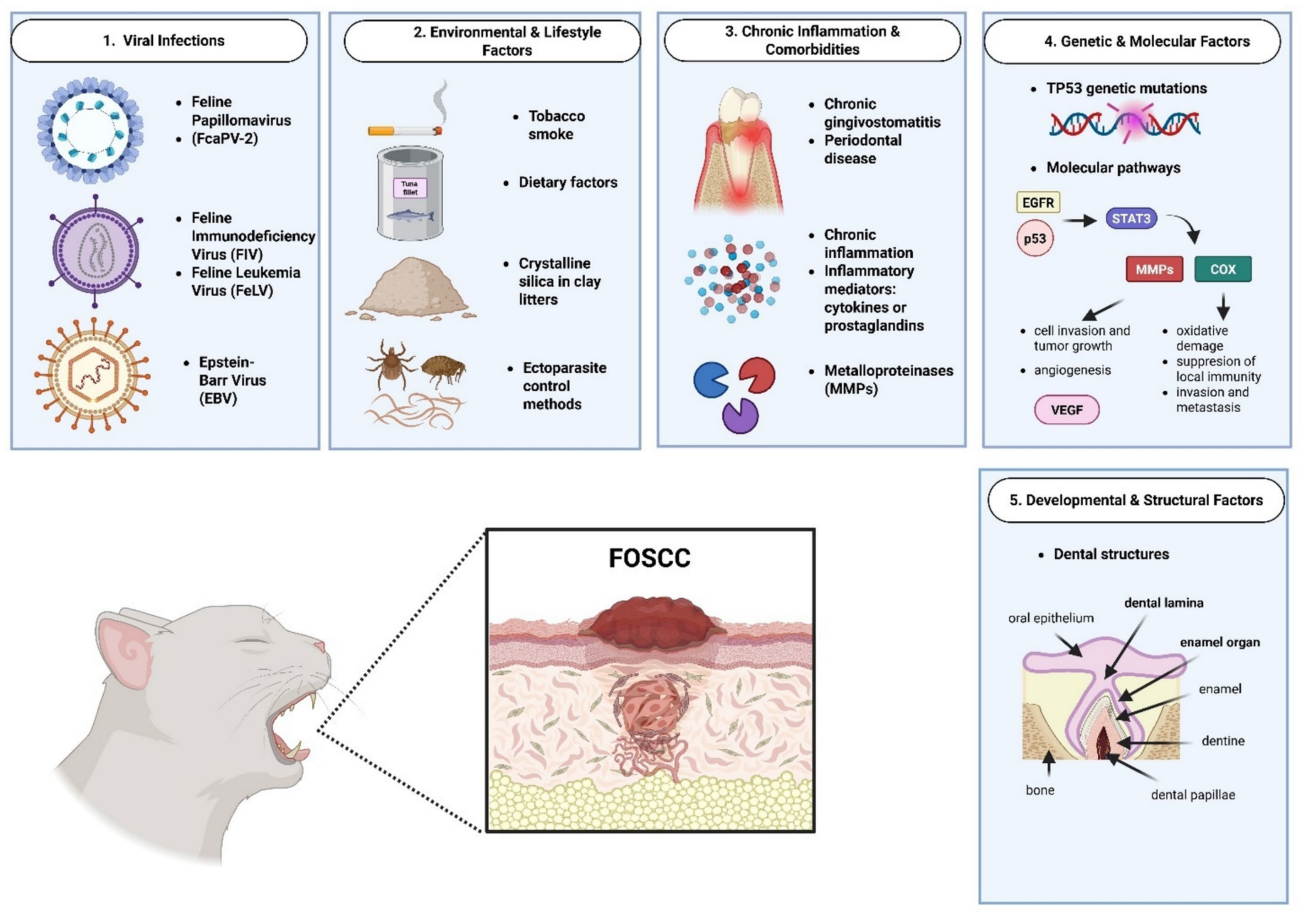
Schematic representation of feline oral squamous cell carcinoma etiologic factors. Created in BioRender. Tutu, P. (2025) https://BioRender.com/zpfsdkn.

Understanding the causes of FOSCC is limited due to few studies, many with small sample sizes or case series (1–3). Using owner-reported data for exposure to ETS may cause bias, as smoking households with affected cats might be underrepresented (1–3). The lack of standard methods for virus detection and missing information about the patient health status may also underestimate the role of infection (9–11, 63). Future research should involve larger, well-controlled studies to better clarify how these factors contribute to FOSCC development (Figure 1).

## 3 Epidemiology

FOSCC is the most common malignant oral tumor in cats, accounting for 46–61.2% of all oral neoplasms in multiple surveys (64, 65). Epidemiological studies have been conducted worldwide, with data collected from the USA, UK, Italy, New Zealand, Slovenia, and Japan, encompassing hundreds of cases (38). Most studies are retrospective and based on histopathological review of biopsy samples, this limitation may influence the possibility in establishing a direct causal relationship between the neoplasia and the potential etiologic factors described in chapter 2 (17, 38, 66–68).

### 3.1 Breed and demographics

Non-pedigree (domestic) cats represent the majority of FOSCC cases, 84–96% in several studies, with domestic shorthair being the most common, purebred cats are less affected, but breeds such as Burmese, Maine Coon, Persian, Chartreux, and Siamese are known to be affected (38, 50, 66–68). The median age at diagnosis typically ranges from 11 to 13.5 years, with reported ranges spanning 1 to 21 years (17, 50, 64, 67, 68). Both sexes are affected, with studies reporting near-equal or slightly higher female representation (17, 50, 67–69).

### 3.2 Anatomical sites and tumor characteristics

FOSCC most frequently arises in the gingiva (mandibular and maxillary), sublingual region, and tongue (17, 68–70). The tongue is more commonly affected in younger cats (mean age 11.9 years), while gingival tumors occur in slightly older cats (mean age 13.6 years). Tumors are often invasive, with frequent bone involvement (osteolysis), especially in maxillary (48%) and mandibular (33%) cases (68, 70).

## 4 Pathology

### 4.1 Histopathology

**Figure 2:**
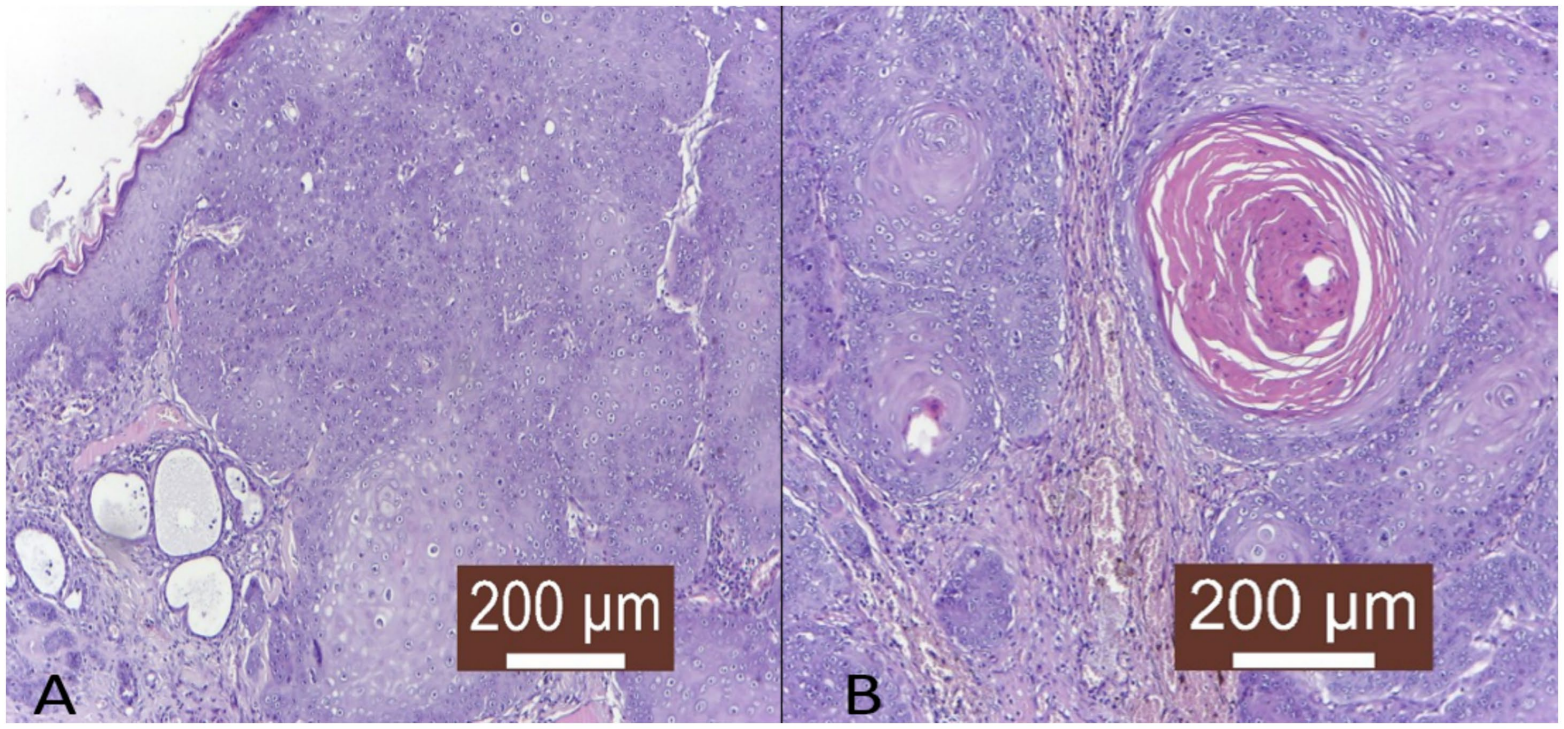
Histopathological representation of feline oral squamous cell carcinoma. **(A)** Irregular, columnar, diffuse tumor infiltration in the deep layers of the skin with the formation of keratin-rich tumor islands (HE X100). **(B)** Tumor islet with high keratin content in the profound layer of the skin (HE X100).

Oral squamous cell carcinomas in domestic animals consist of invasive nests of cancerous epithelial cells penetrating the submucosa, with basaloid outer cells and larger eosinophilic central cells. These tumors show abnormal maturation, keratinization irregularities, and solid masses with intercellular bridges (71). They grow aggressively, often spreading single cells beyond the main tumor (Figure 2A), causing ulceration, inflammation, and sometimes bone invasion with surrounding fibrous tissue. Tumors vary in differentiation, influencing prognosis, and may contain abnormal nuclei, necrosis indicating aggressiveness, multinucleated cells, and lymphocyte infiltration, while stromal fibrosis is generally minimal (71–73).

Several distinct histological subtypes can be identified in FOSCC based on criteria adapted from HNSCC classifications. These subtypes primarily include well-differentiated keratinizing squamous cell carcinoma, poorly differentiated non-keratinizing squamous cell carcinoma, and basaloid squamous cell carcinoma. The well-differentiated subtype is characterized by the presence of keratin pearls and an organized arrangement of squamous cells (Figure 2B), indicative of maintained differentiation. Poorly differentiated variants lack these organized features, often exhibiting a higher degree of pleomorphic cells, which can result in more aggressive clinical behavior (66, 74).

The basaloid variant, although less frequent, presents a distinct histological profile with high cellularity and minimal keratin formation. This subtype can exhibit a pattern similar to that of adenoid cystic carcinoma, which poses diagnostic challenges as it might be misidentified without careful histopathological evaluation (66). The presence of such variants in FOSCC suggests the need for meticulous histological examination and classification, as they correlate strongly with clinical outcomes and prognostic indicators (34, 75).

In both cats and humans, OSCC is the most common oral cancer, characterized by invasive malignant keratinocytes. A key difference is that keratin pearls, common in human OSCC, are rare in cats, reflecting faster progression in felines (74).

### 4.2 Clinical appearance

**Figure 3:**
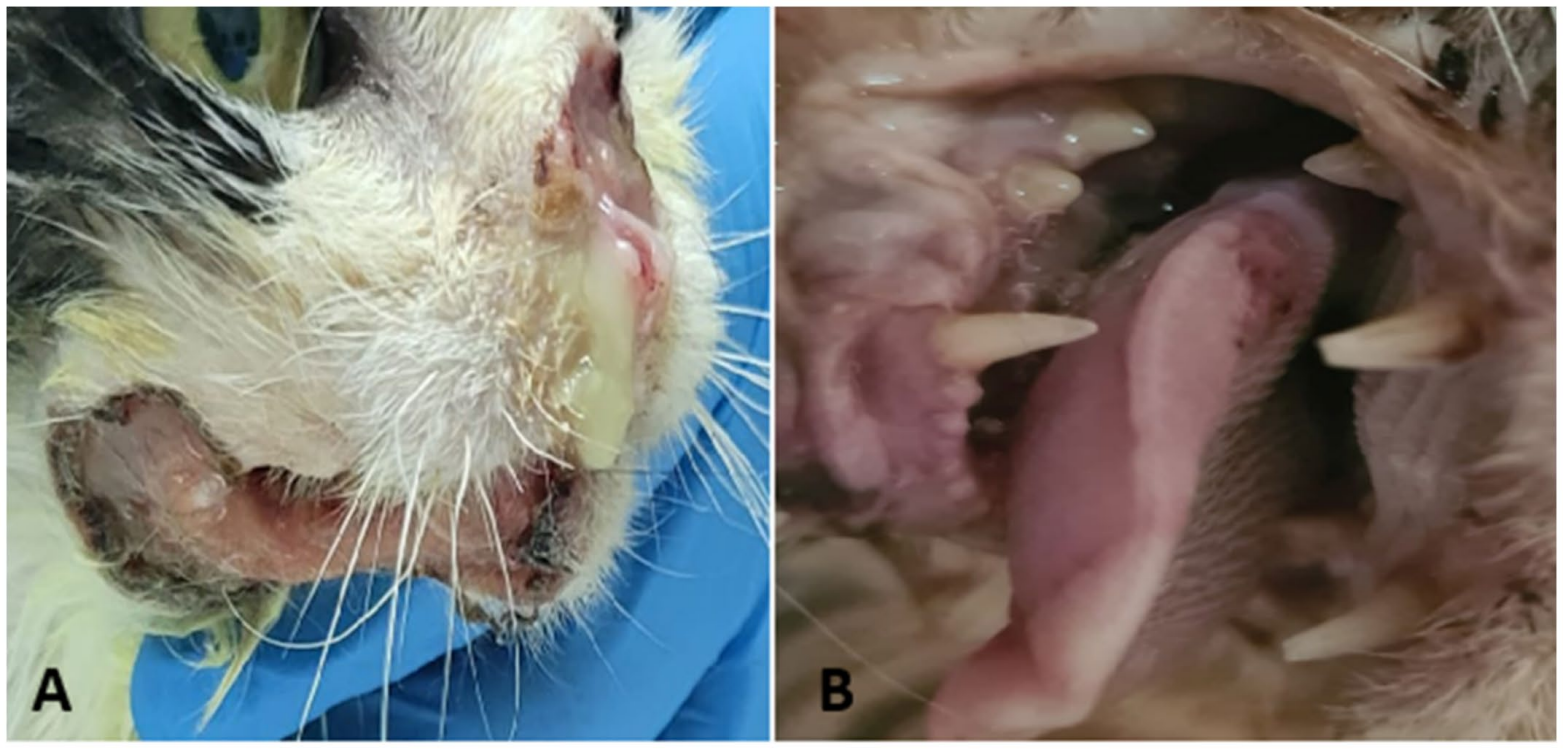
Feline oral squamous cell carcinoma—clinical presentation. **(A)** Ulcerated and infected nasal and mandibular lesion. **(B)** Ulcerated lesion at the base of the tongue.

FOSCC is an aggressive neoplasm that can arise in various locations within the oral cavity, most commonly affecting the mandibular (Figure 3A), maxillary, and sublingual regions (50, 70, 76). Less frequently, tumors develop on the hard palate, soft palate, larynx, pharynx, or lips, although these sites account for less than 2% of cases (70). Regardless of location, SCC tend to be locally invasive and destructive, leading to severe clinical signs.

In the initial stages, FOSCC may appear as a small, raised, fleshy mass or as an ulcerated lesion with little visible proliferation. Despite their relatively subtle appearance on physical examination, these tumors often invade surrounding tissues extensively. Affected cats commonly exhibit nonspecific signs such as reduced appetite, weight loss, lethargy, halitosis, excessive drooling, and decreased grooming. Owners frequently report difficulty in eating, and veterinarians may observe loose or mobile teeth in the affected area. While tooth extraction may temporarily improve appetite, the extraction site often fails to heal, forming a persistent ulcer. Tumors that develop in the sublingual and lingual areas (Figure 3B) can look like a foreign body invading the tongue aggressively. This leads to a firm thickening of the tongue, reduced movement, ulceration, and in severe cases necrosis due to poor blood supply. As these tumors grow, the tongue may stick out of the mouth, which can cause trauma, bleeding, and make eating difficult. Maxillary SCC are highly destructive, spreading into bone and causing bone loss with large lesions. Tumors located toward the back of the upper jaw may interfere with eye movement, while those at the front often cause teeth to become loose or fall out, even if the gums appear healthy (71).

Mandibular SCC show similar signs, including ulceration, loose teeth, and in some cases, new bone formation and bone loss even without visible ulceration. Occasionally, tumors originate within the jawbone itself, resembling intraosseous carcinoma (77).

### 4.3 Biomarkers

The potential role of biomarkers as immunohistochemical markers in feline tumors is a subject of current exploration. A number of significant biomarkers have been evaluated in FOSCC, demonstrating correlations with their counterparts in HNSCC, indicating shared molecular pathways and disease mechanisms.

**Figure 4:**
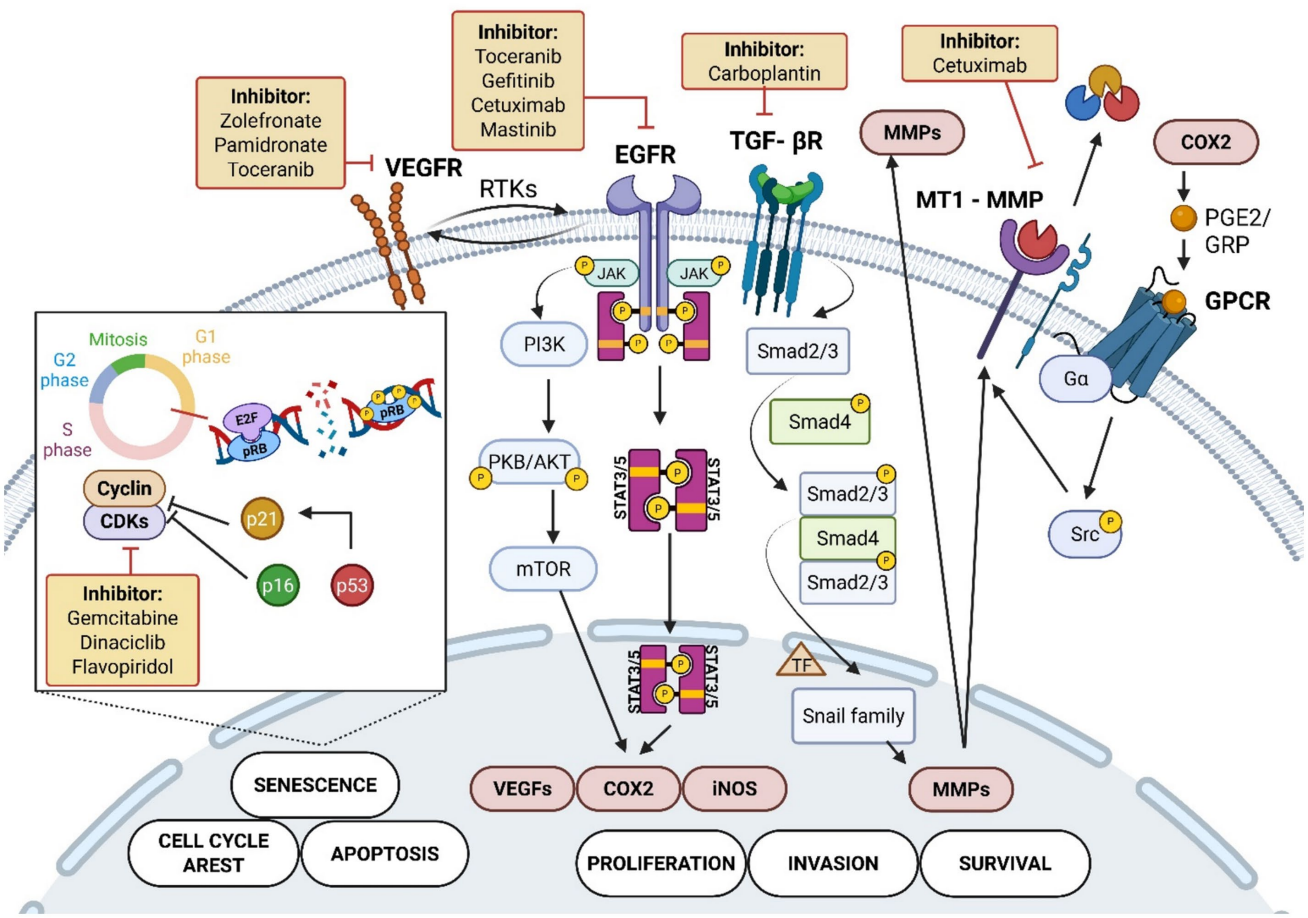
Key biomarkers and targeted treatments in feline oral squamous cell carcinoma. Carcinogens activate receptor tyrosine kinases (RTK) such as VEGFR and EGFR, triggering signaling cascades including JAK/STAT3/5, PI3K/AKT/mTOR, and TGF-*β* receptor/Smad pathways. These pathways regulate cellular processes such as proliferation (COX2, VEGF), survival (MMP), invasion (Snail), apoptosis, senescence, and cell cycle arrest (p53, p16, p21, CDK, Cyclins, E2F, pRB). COX2 activation involves GPCR and Src kinase signaling leading to MMP activity promoting the invasive activity of the tumor. The inset shows cell cycle control through pRB phosphorylation and CDK regulation (5, 8, 25, 102, 139, 144–146). The figure also depicts pharmacological inhibitors targeting these molecules, including Toceranib, Gefitinib, Cetuximab, Gemcitabine, Carboplatin, and others, used in the treatment of FOSCC (4, 46, 91, 96, 115, 116, 124, 125, 128, 135, 142). Created in BioRender. Tutu, P. (2025) https://BioRender.com/3hns4gp.

These biomarkers are classified into several categories, including proliferation markers, epithelial-mesenchymal transition (EMT) factors, immune checkpoints, angiogenesis-related proteins, stromal remodeling elements, genetic alterations, and metabolic regulators. Among these, p53, p16, EGFR, VEGF and COX-2 have been the most studied (2–8, 17, 25, 54, 66, 78–89). However, there are some new markers, particularly Ki67, Bax, Bcl2, Caspase3, NQO1 and TERT, which offers new insights and new perspectives into the FOSCC molecular evolution and targetability (Figure 4; Table 1) (5, 79, 80, 90–92).

**Table 1:** Biomarkers frequently used in the evaluation of feline oral squamous cell carcinoma.

Biomarker category	Biomarker	Activity	Relevance to FOSCC	References
Proliferation markers	Ki-67	Indicator of cell proliferation	Increased expression → aggressive tumor, poor prognosis	(79, 80, 140)
	Cyclin D1	Regulates cell cycle progression	Dysregulation → uncontrolled tumor growth	(81)
	p16	Cell cycle inhibitor	Decreased expression → rapid tumor progression	(5, 11, 29, 30)
	pRb	Tumor suppressor, regulates cell cycle	Inactivated in many cancers, leading to uncontrolled cell growth	(25, 71, 82, 102)
Apoptotic markers	Bcl-2	Inhibits apoptosis	Increased expression → aggressive tumors, treatment resistance	(90)
	Bax	Promotes apoptosis	Decreased expression → accelerated tumor growth	(90)
	Caspase-3	Induces apoptosis	Reduced expression → tumor resistance to cell death	(5, 90, 91)
Angiogenesis and invasiveness markers	CD31 & CD34	Markers of new blood vessel formation	Increased expression → enhanced tumor vascularization	(147)
	VEGF	Vascular endothelial growth factor	Increased expression → angiogenesis and invasiveness	(4–8)
	MMP-2 & MMP-9	Degrade extracellular matrix	Increased expression → promotes invasion and metastasis	(31, 82)
	β-catenin	Involved in cell adhesion	Dysregulation → increased invasiveness	(81, 148)
	CD147	Involved in tumor progression and invasion	Increased expression → associated with metastasis	(149)
	CD146	Cell adhesion and signaling	Upregulated in invasive tumors	(148)
Inflammation and tumor microenvironment markers	COX-2	Mediates inflammation	Increased expression → stimulates tumor progression	(50, 57, 66, 148–150)
	TGF-β	Growth factor and immunosuppressor	Increased expression → promotes immune evasion	(6, 66, 70, 83, 86)
	mPGES-1	Enzyme involved in prostaglandin E₂ synthesis	Elevated expression in adjacent epithelium; potential role in tumor progression	(8)
	TNF-α	Pro-inflammatory cytokine	Elevated levels → contributes to tumor growth and immune evasion	(83)
	IL-6	Pro-inflammatory cytokine	Increased levels → linked to chronic inflammation and tumor progression	(83)
Cancer-associated fibroblast	CAF	Stromal fibroblasts supporting tumor progression	Increased presence → associated with tumor aggressiveness	(86, 87)
	SMA (*α*-SMA)	Myofibroblast marker, involved in stromal remodeling	Increased expression → CAF activation, promoting invasion	(86, 87)
Immune system markers Oxidative stress markers	CD3	T-cell marker	Helps assess immune response in tumors	(66, 86)
	CD4	Helper T-cell marker	Increased expression may correlate with immune infiltration	(66)
	CD79a	B-cell marker	Used for identifying immune responses	(66)
	CD20	B-cell marker	Potential role in immune response and tumor environment	(66, 86)
	CTLA-4	Immune checkpoint inhibitor	Increased expression → immune evasion, potential immunotherapy target	(66)
	STAT3	Transcription factor, regulates gene expression	Increased activation → promotes tumor cell survival and immune suppression	(5)
	8-OHdG	Oxidative stress marker	Increased levels → linked to DNA damage and tumor progression	(92)
	NQO1	Detoxification enzyme, protects against oxidative stress	Upregulated in many tumors, may contribute to chemotherapy resistance	(92)
Metastasis and prognostic markers	PD-1/L1	Inhibits immune response	Increased expression → immune evasion, potential immunotherapy target	(6, 66, 84, 85, 151)
Cell adhesion and prognostic markers	E-cadherin	Regulates cell adhesion	Decreased expression → increased invasiveness and metastasis	(6, 148)
	EGFR	Epidermal growth factor receptor	Increased expression → accelerated tumor proliferation, potential therapeutic target	(49, 78, 79, 135, 140)
	p53	Tumor suppressor	Altered expression → loss of cell cycle control	(2, 3, 17, 25, 53, 54, 83, 88)
	CD44	Cell surface glycoprotein involved in cell adhesion and migration	Overexpression → associated with cancer stem cells and metastasis	(148)
Telomere and senescence markers	TERT	Catalytic subunit of telomerase, maintains telomere length	Overexpression → linked to tumor proliferation and immortalization	(90)
Monocarboxylate transporter	MCT1 MCT4	Involved in tumor metabolism and lactate transport	Altered expression → inhibits tumour growth	(89)

The analysis of biomarkers in FOSCC and their correlation with HNSCC presents the notion of using FOSCC as a valuable naturally occurring model for studying in human’s counterparts. The substantial similarity in biomarker profiles indicates the feasibility of translational research, whereby feline cancer studies could provide insights that inform human medicine, particularly in the context of exploring novel therapeutic strategies and preventive measures (5, 34, 83). Furthermore, given the similarity in the pathways of tumour evolution exhibited by both cancers, interventions targeting shared biomarkers may result in advancements in treatment outcomes across species.

## 5 Prognosis

FOSCC poses significant challenges in veterinary medicine due to its aggressive nature and poor prognosis. Understanding the prognostic factors associated with FOSCC is critical for enhancing treatment strategies and overall outcomes. Much like HNSCC, various biomarkers, clinical indicators and treatment modalities influence the clinical course and therapeutic response of FOSCC.

### 5.1 Treatment modalities

FOSCC carries a poor prognosis, with median survival times (MST) of 44 to 60 days and a one-year survival rate of 5–10% (50, 63, 78, 93). Surgical excision, especially mandibulectomy, can improve survival, with MST reported up to 420 days, though recurrence rates remain high at 38% (94, 95). Traditional radiation and chemotherapy are generally ineffective, accelerated radiation combined with carboplatin has extended MST to around 163 days (96). FOSCC is notably resistant to conventional therapies, with mechanisms of resistance still not well understood (94, 95, 97, 98).

### 5.2 Tumor location

Tumor site influences prognosis. Cats with maxillary SCC tend to have longer survival compared to other oral locations (63). Oropharyngeal SCCs show longer MSTs than sublingual or other sites, possibly linked to differences in cancer-associated fibroblasts (87). Bone invasion does not seem to affect prognosis significantly, reflecting the highly invasive nature of these tumors regardless of histology (17). Metastasis drastically worsens survival, with MST of 24 days for cats with multiple lymph node or distant metastases versus 90 days for non-metastatic cases (63, 93, 94).

### 5.3 Molecular markers

Several molecular markers have prognostic relevance. Diffuse cyclooxygenase-1 (COX-1) expression correlates with longer survival (50). The Ki67 proliferation index shows mixed results: some studies link high Ki67 to worse outcomes, but this is not confirmed in other studies (78, 79). EGFR expression generally does not correlate significantly with survival, though lower survival trends imply potential as a therapeutic target (78, 79). Tumor vascularization assessed by microvessel density (MVD) lacks strong survival correlation, despite higher MVD in tongue tumors (99, 100).

Immunohistochemical markers like p16 associate with longer survival independently of papillomavirus infection (101, 102), while p53 expression is unreliable for prognosis, suggesting diverse oncogenic pathways in FOSCC development (13, 17, 103, 104). Histologic differentiation and invasion patterns have not consistently predicted outcomes (105–107).

FOSCC remains a highly aggressive neoplasm with limited treatment success. The identification of molecular markers may enhance prognostic predictions and guide treatment decisions. Future studies should focus on refining prognostic markers and exploring targeted therapies to improve clinical outcomes for affected cats.

## 6 Therapy

FOSCC remains a challenging disease. Radiation therapy, chemotherapy, and surgery are standard treatments, often used together to control the disease and improve quality of life. Emerging approaches such as metabolic therapy, bisphosphonates, stem cell therapy, immunotherapy, tyrosine kinase inhibitors (TKI), analgesics, and gene therapy are being explored to enhance outcomes (Table 2). Traditional treatments focus on tumor control, while newer therapies attempt to target tumor growth, manage pain, and improve survival, aiming at the diversification and enrichment of the arsenal against this aggressive cancer.

**Table 2:** Therapies reported to be used in FOSCC.

Treatment	Purpose	Survival	Efficiency	References
Stereotactic radiation therapy (SRT)	Radiation	106 days	38.5% response rate	(79)
Accelerated hypofractionated radiation therapy	Radiation	174 days	23% (PFS), 41% (LPFS), 29% (OS)	(108)
Coarse fractionation radiotherapy	Radiation	60 days	Limited palliative effect	(97)
Coarse fractionated megavoltage radiation therapy	Radiation	92-127 days	65% improved QoL	(109)
Accelerated radiation protocol	Radiation	86-298 days	Manageable toxicity	(110)
Gemcitabine + palliative radiotherapy	Chemotherapy	111.5 days	Partial/complete responses in some cases	(115)
Toceranib	Targeted therapy	123 days (treated) vs. 45 days (untreated)	56.5% response rate	(46, 128)
Carboplatin	Chemotherapy	Varies	Enhances radiation efficacy	(96, 116, 117)
USMB-enhanced chemotherapy	Radiation and Chemotherapy	Limited data	Improves tumor perfusion	(118)
IB-DNQ	Chemotherapy	Not specified	Targets NQO1-overexpressing tumors	(92)
Maxillectomy	Surgery	Up to 2 years	83% two-year survival rate	(121)
Radical mandibulectomy	Surgery	712 days	6/8 cats resumed feeding	(152)
MD-1 Therapy	Metabolic therapy	Limited data	Reduces tumor growth	(89)
Zoledronate + Meloxicam	Bisphosphonates	Not specified	Inhibits osteolysis, angiogenesis	(4, 124)
Pamidronate	Bisphosphonates	Not specified	Stable disease in select cases	(125)
Microbrachytherapy	Internal radiation	113-296 days	55% local response	(111)
Stem cell therapy	Cellular therapy	<1 month	Temporary symptom relief	(128)
L-NDDP	Chemotherapy	Poor survival	No tumor response	(119)
LAK cell transplantation	Immunotherapy	Not extended	Safe but ineffective	(129)
ECEA	Ablation	Not viable	Transient tumor reduction	(120)
Tyrosine kinase inhibitors	Targeted therapy	Not specified	Dual inhibition of FOSCC pathways	(126, 135)
DFMO	Metabolic therapy	Not specified	Reduces tumor polyamine levels	(122, 123)
Gene therapy	Genetic targeting	Not specified	Partial response in one cat	(127)
*Viscum album* extracts	Plant-base therapy	Not specified	Induce apoptosis and cell cycle arrest	(153)
Cold atmospheric plasma	Reactive oxygen and nitrogen	Not specified	Effective antitumor activity in SCC tumor	(154)

### 6.1 Radiation therapy

Radiation therapy for FOSCC demonstrates clinical feasibility through various protocols that offer symptom relief and tumor control, but outcomes and tolerability vary considerably. Stereotactic Radiation Therapy (SRT) provides rapid symptom improvement with an overall response rate of 38.5% and median survival around 106 days. However, factors such as high tumor microvascular density and keratinization negatively impact survival, and complications like mandibular fractures can impair quality of life (80). Accelerated hypofractionated radiation (4.8 Gy × 10) shows promising tumor responses and extended progression-free and overall survival with generally manageable mucositis and some late effects, although long-term toxicity remains a concern (108).

Coarse Fractionation Radiotherapy (8 Gy × 3) offers limited palliation and shorter median survival (60 days) but poses substantial risks including mucositis, pain, and dysphagia, which can affect clinical tolerability (97). A similar coarse fractionated megavoltage protocol (24–40 Gy in 3–4 fractions) improves quality of life in a majority of cats, with median survival ranging near 92 days (109). The accelerated protocol (3.5 Gy × 14 over 9 days) is moderately tolerable and achieves a median survival of 86 days, while cats achieving complete response may survive substantially longer (110). Microbrachytherapy with holmium-166 microspheres shows encouraging local control rates (55%) and minimal side effects, allowing for less extensive surgery in some cases and improved survival in responders (111).

Despite initial radiosensitivity, FOSCC frequently develops radioresistance over time, driven by mechanisms such as enhanced DNA repair, cancer stem cell activation, EMT, and tumor microenvironment changes. These adaptations reduce long-term treatment efficacy and contribute to the overall poor prognosis (112–114). Current limitations include variable survival benefits, toxicities affecting quality of life, and the inevitability of radioresistance. Future directions emphasize the development of multimodal strategies that integrate radiation therapy with surgery, systemic treatments, or molecular targeted therapies to overcome resistance and improve clinical outcomes (112–114).

### 6.2 Chemotherapy

Chemotherapy shows clinical feasibility with several agents explored, though overall efficacy remains limited and survival benefits modest. Low-dose gemcitabine combined with palliative radiotherapy achieves partial or complete responses in some cats, with a median survival time of 111.5 days (115).

Carboplatin acts as a radiosensitizer in accelerated radiation protocols, particularly benefiting tonsillar SCC with manageable toxicity and modest antitumor activity (96, 116, 117). Ultrasound and microbubble-enhanced chemotherapy (USMB) using bleomycin is a clinically feasible and safe approach to improve drug delivery and tumor perfusion but has shown limited clinical efficacy in cats (118). The novel agent IB-DNQ, targeting NQO1 to generate cytotoxic reactive oxygen species, presents promising targeted therapeutic potential (92). Conversely, liposomal cisplatin (L-NDDP) proved ineffective, yielding no tumor responses and poor survival despite acceptable toxicity (119). Ethyl Cellulose-Ethanol Ablation (ECEA), which combines chemotherapy with localized electric pulses to retain ethanol intratumorally, produced transient tumor shrinkage but poor functional outcomes in lingual and sublingual SCC, limiting its applicability to these sites (120).

Limitations of chemotherapy include generally modest survival improvements, inconsistent tumor responses, and treatment-related toxicities that affect quality of life (118–120). Future directions should focus on refining multimodal protocols integrating chemotherapy with radiation, surgery, and novel targeted agents to enhance efficacy. Continued research into innovative drug delivery systems and molecularly targeted therapies is essential to overcome current therapeutic challenges and improve prognosis in FOSCC.

### 6.3 Surgical interventions

Surgical interventions include maxillectomy and mandibulectomy. These are aggressive procedures but offer a potential curative approach if the tumor is localized and able to extract (94, 121).

Maxillectomy is an effective treatment for FOSCC, achieving good local tumor control and extended survival times. The procedure includes various techniques, such as unilateral rostral, bilateral rostral, segmental, caudal, and total unilateral maxillectomy. While intraoperative complications occur in 16.7% of cases, postoperative complications are more common, with hyporexia and incisional dehiscence affecting 20% of cats. Despite these challenges, survival rates are promising, with a two-year survival rate of 83% for FOSCC cases. Poor prognostic factors include a high mitotic index, the need for adjuvant chemotherapy, and local recurrence, which significantly impact survival (121).

Radical mandibulectomy is another aggressive surgical approach for managing extensive FOSCC. The procedure involves removing 75 to 90% of the mandible, necessitating feeding tube placement in all cases. While some cats experience local recurrence, others achieve long-term survival, with some living beyond 1 year. The mean estimated survival time following mandibulectomy is 712 days. Importantly, six out of eight cats were able to resume independent food intake postoperatively. With appropriate perioperative supportive care, radical mandibulectomy is a viable option for treating extensive feline oral neoplasia and can result in prolonged survival for selected patients (94).

Future directions should focus on minimizing surgical morbidity, improving perioperative care, and integrating multimodal therapies to address local recurrence and metastatic disease. Investigating less invasive techniques or adjunct treatments to enhance surgical success and quality of life is warranted.

### 6.4 Metabolic therapy

Metabolic therapy represents a clinically feasible and innovative approach by targeting cancer-specific energy and nutrient metabolism. MD-1 therapy disrupts glycolytic and mitochondrial metabolism, particularly oxidative phosphorylation (OXPHOS), effectively killing FOSCC cell lines and reducing tumor growth in both subcutaneous and orthotopic models. These promising *in vitro* and *in vivo* findings position MD-1 as a potential novel treatment for FOSCC and possibly HNSCC (89).

Another metabolic strategy targets polyamine synthesis using 2-Difluoromethylornithine (DFMO), which lowers tumor polyamine levels essential for proliferation. While DFMO monotherapy can reduce tumors, notable toxicities such as ototoxicity and subclinical thrombocytopenia present limitations that require further optimization (122, 123). The combination of DFMO with MQT 1426 is feasible and safe, yielding modest clinical benefits like stable disease or tumor regression; however, dosing adjustments are necessary to reduce vestibular toxicity.

Although metabolic therapies show promise by exploiting tumor-specific metabolic vulnerabilities, challenges remain regarding toxicity and optimal dosing protocols. Future directions should involve refining such metabolic interventions, integrating them with conventional therapies, and expanding translational research to improve outcomes for FOSCC and related human cancers.

### 6.5 Bisphosphonates

Bisphosphonates are useful for managing bone-invasive FOSCC. They work by blocking osteoclastic bone resorption and angiogenesis, which helps reduce pain. Zoledronate can slow tumor growth and reduce bone damage. It lowers levels of serum VEGF and C-terminal telopeptide (CTx), markers linked to tumor activity. When given with meloxicam, zoledronate is well-tolerated. Meloxicam helps slow tumor growth, while zoledronate prevents bone breakdown (4, 124).

Pamidronate is another bisphosphonate that also blocks bone resorption and blood vessel growth. A small study in eight cats with bone-invasive cancers, including FOSCC, found pamidronate to be safe and feasible. It showed modest benefits, like stabilizing the disease in some cats. However, no direct tumor shrinkage was seen. Still, pamidronate’s ability to inhibit tumor cells in lab tests and ease bone-related symptoms supports further research (125).

Bisphosphonates mainly offer palliative benefits rather than curing the disease. More studies are needed to improve their use, possibly combining them with other treatments. This could help improve quality of life and clinical outcomes in cats with bone-invasive FOSCC.

### 6.6 Emerging therapies

Emerging therapies, such as TKI, stem cell therapy and gene therapy were used in FOSCC patience as alternative treatments.

Mastinib, a TKI, is effective at slowing cancer cell growth. This approach targets important pathways and works in both cats and dogs (126). Gene therapy using TBG-RNAi-fCK2αα’ is safe and shows some signs of shrinking tumors (127). Both treatments appear feasible and deserve further trials (126, 127). Toceranib, a TKI, offers modest efficacy, extending median survival to 123 days compared to 45 days in untreated cats, with a biological response rate of 56.5%. Improved outcomes are noted when combined with NSAIDs, yet long-term survival is still poor (128).

Stem cell therapy using feline umbilical cord MSCs can reduce symptoms for a short time (128). However, the benefits are temporary, and disease quickly worsens. Immunotherapy with lymphokine-activated killer (LAK) cells is safe even in older cats, but it has not been shown to extend survival or slow cancer (129).

All therapies have limited or inconsistent effects. Stem cell treatment only relieves symptoms briefly without improving survival (128). Immunotherapy is well tolerated but lacks proof of effectiveness (129). Gene therapy needs better dosing and more reliable results (127). The TKI data come from small studies and need stronger evidence from larger trials (126).

More research with larger, prospective studies is needed. Combining TKI with other treatments might improve results. Gene therapy should be fine-tuned for dosing and timing. Immunotherapy approaches must identify better targets and boost immune responses. Using multiple therapies together could offer better tumor control and longer survival. Developing biomarkers will help make treatments to individual cats for better outcomes.

## 7 Future perspectives

### 7.1 New areas of interest

Research in FOSCC is advancing by focusing on several promising areas. Tumor mutational burden (TMB), defined as the number of somatic mutations per megabase in tumor DNA, is emerging as a biomarker to predict response to immune checkpoint inhibitor therapy. FOSCC shows a high TMB (>5.0), similar to HNSCC, suggesting potential responsiveness to future immune checkpoint therapies (83, 130).

Genomic studies have identified polyamine-related signatures that influence tumor metabolism and the microenvironment, offering new therapeutic targets (131). Additionally, EMT-related genes such as SNAI1, TWIST1, ZEB1, ZEB2, and mesenchymal markers FN1, VIM, and CDH2 are enriched in FOSCC and contribute to metastasis (83). Although immune checkpoint inhibitors like PD-L1 and CTLA-4 are not yet available for FOSCC treatment, they remain promising candidates for immunotherapy trials (6, 66, 83–85). Metabolic targeting approaches, including dual MCT1/MCT4 inhibitors and pathways regulated by hypoxia-inducible factors such as HIF-1α, are being explored as potential therapies (89).

The focus on molecular and metabolic factors, such as high TMB and EMT gene expression, that highlight tumor vulnerabilities, appears to be effective in the FOSCC research. These findings support immune checkpoint inhibitors and metabolic targeting as promising therapeutic strategies because they address key mechanisms driving tumor growth and spread.

### 7.2 New therapeutic frontiers

FOSCC is an aggressive malignancy with poor prognosis and novel treatment approaches are challenging. Recent advances are related to innovative strategies enhancing therapeutic outcomes.

#### 7.2.1 Electrochemotherapy (ECT)

Electrochemotherapy (ECT) has emerged as a promising localized treatment for FOSCC. Studies have demonstrated its efficacy, particularly in combination with bleomycin. One study reported an 81.8% complete response rate in superficial SCC lesions, with some responses lasting over 3 years, highlighting its durability and tolerability (132). Another study showed that ECT with bleomycin significantly outperformed bleomycin alone, achieving an 89% overall response rate and a median progression-free survival of 30.5 months, compared to 3.9 months in the control group (133). These findings underscore ECT’s potential for managing advanced SCC in critical areas like the head.

#### 7.2.2 Targeted molecular therapies

Targeted molecular therapies for FOSCC include EGFR-targeted agents, telomerase inhibitors, nanobody-targeted photodynamic therapy, and bone-targeted treatments, each designed to interfere with specific pathways involved in tumor growth and progression.

EGFR is a key driver in epithelial cancers due to its frequent overexpression or mutation, which leads to persistent activation of signaling pathways that promote tumor cell proliferation, survival, invasion, and metastasis, making it a critical molecular target for therapies such as TKI and monoclonal antibodies (134–139).

Cetuximab, an anti-EGFR monoclonal antibody used as therapeutic agent against HNSCC, has demonstrated efficacy in FOSCC cell lines. It inhibits EGFR activation and downstream signaling pathways such as Akt, reducing proliferation, promoting apoptosis, and impairing invasion by downregulating matrix metalloproteinases (MMP-2/-9) and EMT markers (82, 135). These findings suggest that Cetuximab could be a valuable addition to feline cancer therapy.

Gefitinib, an EGFR TKI, has been shown to suppress cell proliferation and migration in FOSCC. Resistance to gefitinib can occur, not due to mutations in its kinase domain. RNA interference (RNAi) targeting EGFR has demonstrated potential in overcoming this resistance and exhibits an additive effect when combined with radiation therapy (140).

#### 7.2.3 Immunotherapy

The potential of immunotherapy in FOSCC treatment is being explored, with particular focus on immune checkpoint inhibitors. Nivolumab, an anti-PD-1 antibody approved for recurrent or metastatic HNSCC in humans, has demonstrated significant survival benefits over standard therapies. While its application in FOSCC is under investigation, its success in human oncology suggests a promising translational opportunity (141).

#### 7.2.4 Chemotherapeutics

Several chemotherapeutic agents used for other cancers have demonstrated efficacy against FOSCC. Methotrexate, actinomycin D, and CDK inhibitors such as dinaciclib and flavopiridol have shown strong anti-proliferative effects on FOSCC cell lines while sparing normal fibroblasts. These agents induce apoptosis and alter cell cycle progression, making them viable candidates for further clinical trials in feline oncology (91, 142). Additionally, methotrexate’s established efficacy in HNSCC supports its potential use in FOSCC (143). The utilization of these agents in the human oncology field, accompanied by their results in FOSCC cell lines, makes them promising targets in FOSCC future therapy.

## 8 Conclusion

FOSCC remains the most common and aggressive oral malignancy in cats, posing significant challenges for both diagnosis and treatment. Its multifactorial etiology highlights the complexity of this disease and the need for a holistic approach to both research and clinical management. Despite advances in our understanding of the molecular and cellular mechanisms of FOSCC, the prognosis for affected cats remains poor, with median survival times rarely exceeding few months.

Recent research has revealed some promising opportunities for improving the diagnosis, prognosis, and treatment of FOSCC. The identification of key biomarkers, such as Ki-67, Cyclin D1, Bmi-1, and EMT-related proteins, has improved our ability to diagnose and prognosticate FOSCC, while studies on genetic mutations and molecular pathways (including TP53, COX, STAT3, EGFR, and VEGF) have provided valuable insights into tumor behavior and potential therapeutic targets. New areas of interest, such as TMB and immune checkpoint molecules, suggest that immunotherapy and metabolic targeting may play a future role in treatment.

FOSCC is characterized by its rapid local invasion, and destructive nature, often leading to severe oral discomfort and a marked decline in quality of life. While surgical excision offers the best chance for prolonged survival, it is rarely feasible due to the tumor’s location and extent at diagnosis. Traditional therapies, including radiation and chemotherapy, have shown limited efficacy, though novel protocols and combination treatments show some promise.

Progress in the management of FOSCC will depend on early detection, larger and better epidemiological studies, and applying molecular discoveries into practical clinical tools. The integration of advanced diagnostics, personalized medicine, and innovative therapies holds the potential to improve both survival and quality of life for affected cats. Ongoing collaboration among researchers, clinicians, and pet owners will be essential to stimulate innovation and ensure that scientific advances benefit feline patients in tangible ways.
